# Reversible switching from fluorescence to room temperature phosphorescence amplified by exciton-vibration coupling through pressure-induced tiny packing changes†

**DOI:** 10.1039/d4sc02867h

**Published:** 2024-08-07

**Authors:** Yangyang Cao, Zhenzhen Xu, Xinyuqi Zhao, Yong Yang, Haoran Liu, Pingyang Wang, Miao Yu, Hao Li, Hongbing Fu

**Affiliations:** a Beijing Key Laboratory for Optical Materials and Photonic Devices, Department of Chemistry, Capital Normal University Beijing 100048 P. R. China xuzhenzhen@cnu.edu.cn hbfu@cnu.edu.cn

## Abstract

Investigating the impact of exciton–vibration coupling (EC) of molecular aggregates on regulating the excited-state dynamics and controlling room temperature phosphorescence (RTP) emissions is crucial and challenging. We designed and synthesized ArBFO molecules and cultured two crystals with similar molecular packing and completely different luminescent mechanisms from B-form fluorescence to G-form RTP. The mechanism study combining measurement of photophysical properties, time-resolved fluorescence analysis, X-ray diffraction analysis, and theoretical calculations shows that tiny changes in molecular stacking amplify the EC value from B-form to G-form H-aggregates. The larger EC value accelerates the ISC process and suppresses the radiative singlet decay. Meanwhile, the stronger intermolecular interaction restricts non-radiative transitions. All of these facilitate green RTP emission in G-form aggregates. When treated with pressure–heating cycles, the transformation between B-form and G-form aggregates leads to a reversible blue fluorescence/green RTP switch with good reproducibility and photostability. Moreover, their potential in multi-level information encryption and anti-counterfeiting application has been well demonstrated. The results of this research deepen the understanding of the effect of aggregation on the luminescence mechanism and provide a new design guidance for developing smart materials with good performance.

## Abstract 2

The regulation of fluorescence and RTP properties through manipulating exciton–vibration coupling through pressure-induced tiny packing changes was realized.

## Graphical abstract

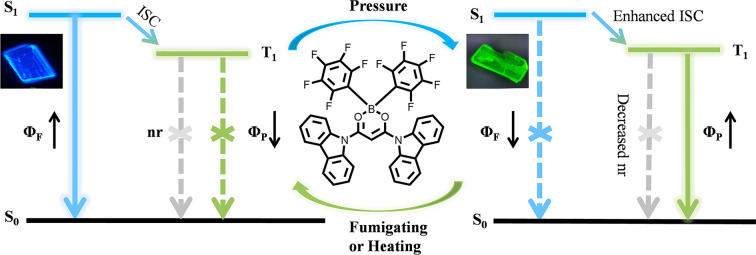

## Introduction

High performance purely organic luminescent materials have been widely investigated due to their unique photophysical properties and their potential applications in sensing,^1^ biological imaging^2^ and light-emitting devices.^3^ For these applications, organic semiconductors can generate singlet-exciton-based fluorescence,^4^ triplet-exciton-based phosphorescence or thermally activated delayed fluorescence.^5^ In basic photophysical processes, photon absorption results in an organic semiconductor being in the lowest excited state S_1_. Then, S_1_ relaxes to the ground state S_0_, *via* a non-radiative or radiative process, emitting a photon and generating fluorescence. Phosphorescence is generated through the intersystem crossing (ISC) from S_1_ to T_1_, and then the radiative decay process from T_1_ to S_0_ which is competitive with non-radiative decay and the oxygen- or moisture-induced process. It is very meaningful to regulate excited-state dynamics to tune room-temperature phosphorescence.

Over the past few decades, significant efforts have been devoted to the development of room-temperature phosphorescent materials with excellent performance.^6^ In order to enhance the ISC efficiency, various molecular design strategies have been proposed. These strategies include enhancing the spin–orbit coupling (SOC) by leveraging the heavy-atom effect and the EI-Sayed rule,^7^ as well as narrowing the singlet–triplet energy gap (Δ*E*_ST_) through introducing donor and acceptor subunits to promote intramolecular charge transfer (CT).^8^ Furthermore, to address unfavourable triplet loss channels, several methodologies are commonly employed. These methodologies include host–guest complexation, matrix rigidification, and ionic crystal formation, aimed at enforcing molecular rigidification for effectively suppressing non-radiative decay and blocking oxygen for minimizing the quenching process.^9^ Overall, these approaches are employed to fine-tune the luminescence characteristics of individual molecules.^10^

Although the emissive behaviours are closely related to the electronic nature of the molecular structure, the exciton–vibration coupling (EC) of the molecular packing mode also affects the properties to a large extent.^11^ To the best of our knowledge, the pivotal role of arrangement of molecules remains less explored in the area of phosphorescence emission. The exploration of the role of molecular aggregates in controlling RTP emissions is of great significance and challenging.

In recent years, our research group has been focusing on investigating the influence of molecular aggregates on luminescent properties.^12^ In previous work, the change of the arrangement pattern through external stimuli such as pressure or heat has been realized and a novel excitonic piezoceramic luminescence mechanism has been developed.^13^ The slight change in packing arrangement might amplify the according EC effect in molecular assemblies, resulting in distinct luminescence switching behaviour. Based on these findings, we speculate that the EC effect in molecular aggregates could be harnessed to develop novel high-performance organic RTP materials and responsive smart materials that can be controlled by external stimulation. This research has the potential to pave the way for exciting advancements in the field of luminescent materials.

**Fig. 1:**
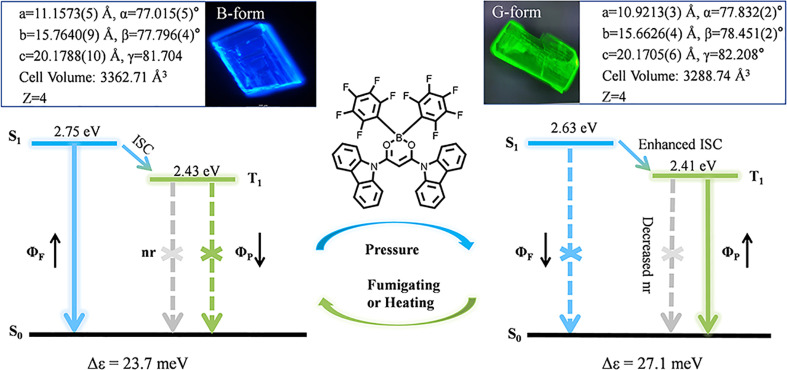
The PL image and crystal unit cell parameters of B-form and G-form crystals. The molecular structure of ArBFO. Proposed energy level structure of B-form and G-form aggregates during the pressure–fumigating or pressure–heating process.

In this paper, we realized the regulation and transition of fluorescence and RTP properties through manipulating exciton–vibration coupling through pressure-induced tiny packing changes, revealing the relationship between crystal stacking and triplet excitons. As shown in Fig. 1, we designed and synthesized a new molecule 9,9′-(2,2-bis(perfluorophenyl)-2H-1λ^3^,3,2λ^4^-dioxaborinine-4,6-diyl)bis(9H-carbazole), named as ArBFO. Two kinds of polymorph crystals emitting blue fluorescence (B-form) and green RTP (G-form) with high quality were obtained under different growth conditions. The mechanism study combining measurement of photophysical properties, time-resolved fluorescence analysis, X-ray diffraction analysis and theoretical calculations reveals that both B-form and G-form crystals belong to the same space group with almost the same cell parameters and similar molecular packing, advocating H-type aggregation in both cases. The tiny reduced cell parameter and the cell volume (about 2% reduction) shown in Fig. 1 induce a 14% increase of the EC value form 23.7 meV (B-form) to 27.1 meV (G-form) and an enhancement of intermolecular interactions. The larger EC value induces a decreased emission oscillator strength which suppresses the radiative singlet decay. Subsequently, a smaller Δ*E*_ST_ value of 0.22 eV for G-form crystals compared to the value of 0.32 eV for B-form crystals facilitates the ISC process. And the stronger intermolecular interaction restricts non-radiative transitions in G-form crystals. The enhanced ISC process and further restricted non-radiative transitions facilitate green RTP emission in G-form crystals. When treated with pressure–heating cycles, the transformation between B-form and G-form aggregates leads to a reversible blue fluorescence-green RTP switch with good reproducibility and photostability. Moreover, their potential application, especially in multi-level information encryption and anti-counterfeiting areas, has been well demonstrated.

## Results and discussion

### Synthesis of ArBFO

ArBFO was synthesized using a slightly modified version of the procedure reported in the literature.^8*e*^ The yield of ArBFO is 63%, The chemical structure of ArBFO was characterized using ^1^H NMR, high-resolution mass spectrometry and ^13^C NMR (Fig. S1–S4†).

### Photophysical properties

**Fig. 2:**
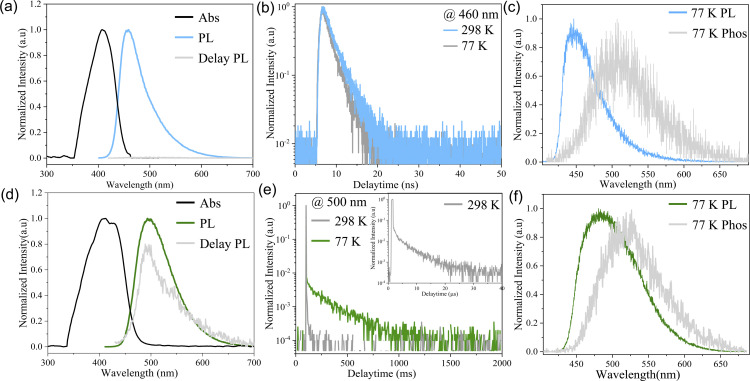
(a and d) Steady-state absorption and PL spectra of B-form (a) and G-form (d) crystals at RT. (b and e) Decay curves of B-form (b) and G-form (e) crystals at RT and 77 K. (c and f) Normalized PL and phosphorescence spectra of B-form (c) and G-form (f) crystals at 77 K.

Two kinds of polymorph crystals with high quality were obtained through slowly evaporating a mixed solution of DCM/cyclohexane and DCM/methanol, respectively (ESI†). Under UV excitation (330–380 nm), these two polymorphic forms exhibit blue and green emissions, as shown in the insets in Fig. 1. Fig. 2d and f, and ESI S5† depict the absorption spectra and photoluminescence (PL) spectra of blue crystals (CCDC: 2336022) and green crystals (CCDC: 2336023). The diffuse reflectance absorption spectrum of B-form crystals exhibited a maximum peak at 410 nm. The G-form crystals exhibit a slight redshift absorption with the maximum peak around 416 nm. The emission spectrum of B-form crystals exhibits a maximum at 460 nm without no delayed component. In comparison, the PL of G-form crystals shows its maximum value at 499 nm. It can be seen from Fig. 2d that crystals manifest a significant delayed emission behaviour, and the delayed PL spectra (grey line) are almost similar to the prompt PL (green line), indicating that the prompt PL and delayed PL originate from the same emission excited state.

In order to understand the PL origin of the two crystals, we measured the temperature-dependent PL spectra and time-resolved PL spectra as shown in Fig. 2 and S6–S9†. For comparison, Table 1 also summarizes their relevant photophysical parameters. As shown in Fig. 2b, the PL spectrum of B-form crystals exhibits a mono-exponential decay of 2.4 ns at 298 K. With the decrease of temperature to 77 K, the spectrum remains basically unchanged as shown in Fig. S6a.† All the results verified that the PL of blue crystals is attributed to fluorescence.

**Table 1:** Photophysical parameters of ArBFO monomers in solid-state aggregation

Sample	Abs^a^ (nm)	*λ*_F_ a (nm)	*λ*_Phos_ a (nm)	*τ*_F_ a (ns)	*τ*_Phos_ a (us)	*Φ* a (%)	*λ*_F_ b (nm)	*λ*_Phos_ b (nm)	*τ*_F_ b (ns)	*τ*_Phos_^[^ b ^]^ (ms)	*E*_S_/*E*_T_^b^ (eV)	Δ*E*_ST_^c^ (eV)
B-form	410	456	—	2.40	—	45	450	510	2.04	269	2.75/2.43	0.32
G-form	∼416	—	496	—	4.85	24	—	515	—	252	2.63/2.41	0.22

a PL spectra and lifetime measured at 298 K.

b PL spectra and lifetime measured at 77 K.

c Δ*E*_ST_ = *E*_S_ − *E*_T_.

In sharp contrast, G-form crystals exhibit a significant RTP phenomenon. As shown in Fig. S6b,† as the temperature decreases, the PL spectrum of the green crystal remains unchanged, with a maximum value of around 499 nm. At the same time, the PL intensity at 77 K is significantly enhanced by about 6 times compared to that at 298 K. In comparison with B-form crystals, the PL spectrum of G-form crystals exhibits a longer lifetime of 4.85 us at 298 K as shown in Fig. 2e. Furthermore, the PL lifetime significantly increases to 252 ms with decreasing temperature to 77 K. Together with the enhanced PL intensity with decreasing temperature, the fact that the PL of G-form crystals mainly originates from phosphorescence has been confirmed (Fig. S9†).

To understand the different PL mechanisms of B-form and G-form crystals at room temperature, we have studied the steady-state and delay spectra of B-form and G-form crystals at 77 K using a streak camera (Fig. 2c and f). According to the absorption band edge of the absorption spectra and the maximum emission wavelength of the phosphorescence spectra at 77 K, the energy levels of S_1_ and T_1_ states can be estimated as shown in Table 1. For the B-form crystal, the singlet energy level was 2.75 eV, and the triplet energy level was 2.43 eV, indicating a small Δ*E*_ST_ value of 0.32 eV. Notably, the energy levels of S_1_ and T_1_ states of G-form crystals were 2.63 eV and 2.41 eV, respectively, also indicating a small Δ*E*_ST_ value of 0.22 eV. The small Δ*E*_ST_ values facilitate the ISC process from S_1_ to T_1_ both in G-form and B-form crystals, resulting in the persistent phosphorescence of G-form and B-form crystals at 77 K as shown in Fig. S7 and S8.†

### Crystal data analysis and theoretical calculations

**Fig. 3:**
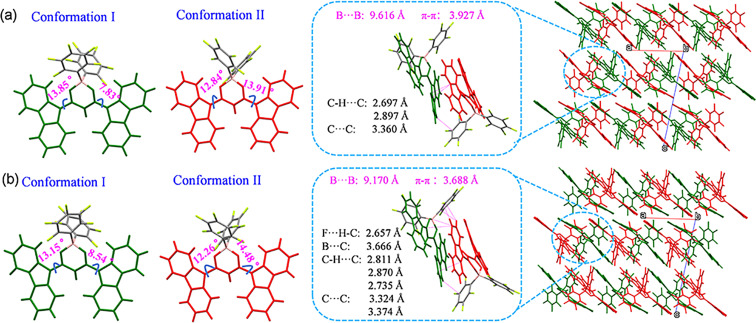
(a and b) Single crystal molecular structures, side view of the dimer and the molecular arrangement of B-form (a) and G-form (b) crystals.

In order to better understand the different luminescence mechanisms of the two crystals, the structural analysis from the molecular conformations and packing modes of the two polymorphs was performed. Both the B-form and G-form crystals belong to the triclinic space group *P*1̄ with *Z* = 4 (Fig. 1 and Table S1†) with almost the same cell parameters and similar molecular packing as shown in Fig. 3a and b. The slightly reduced cell parameters shown in Fig. 1 and Table S1† and the increase of cell volume from 3362.71 Å^3^ to 3288.74 Å^3^ mean a reduced intermolecular distance from B-form to G-form crystals, resulting in a stronger intermolecular interaction, which helps to explain the slightly red-shifted absorption spectra with the maximum absorption peak at 410 nm of B-form crystals to around 416 nm of G-form crystals.

In both crystals, there are two different conformations of ArBFO molecules, respectively, named as I and II, as shown in Fig. 3a. All the conformations are almost planar with a small dihedral angle (*θ*) between carbazole and β-diketonate units (D–A) in the range of 7.83°–14.48°. It can be seen from the single crystal structures in Fig. 3 that two adjacent ArBFO molecules I and II are antiparallel with the β-diketonate unit group and carbazole group on the opposite sides, forming a face-to-face antiparallel dimer structure through the weak intermolecular interaction both in B-form and G-form crystals. Then, these dimers serve as actual building blocks responsible for the formation of G-form and B-form crystals. In the B-form crystal, the dimer displays a B⋯B distance of 9.616 Å, a π–π distance of 3.927 Å and an overlap of 10% as shown in Fig. 3 and S10a†, which indicates a weak π–π interaction. In addition, there are only two types of C–H⋯C (*d*_C–H⋯C_ = 2.697 Å, 2.897 Å) and one type of C⋯C (*d*_C⋯C_ = 3.360 Å) intermolecular interactions in the dimer of B-form crystals. In comparison, the dimer of G-form crystals displays a decreased B⋯B distance of 9.170 Å, a decreased π–π distance of 3.688 Å and an increased overlap of 13% as shown in Fig. S10b.† Moreover, there are more intermolecular interactions in the dimer of G-form crystals, including three types of C–H⋯C (*d*_C–H⋯C_ = 2.811 Å, 2.870 Å, 2.735 Å), two types of C⋯C (*d*_C⋯C_ = 3.324 Å, 3.374 Å), a new type of F⋯H–C (*d*_F⋯H_–_C_ = 2.657 Å) and a new type of B⋯C (*d*_B–C_ = 3.666 Å). All the results indicate stronger intermolecular interactions, which will restrict the intramolecular motions effectively and reduce non-radiative transitions, contributing much to the RTP of G-form crystals.

It is widely believed that the optical and electronic properties of solid-state organic luminescent materials are significantly influenced by the EC, which largely depends on the arrangement of molecules. We use the energy splitting method^14^ to calculate the EC value between adjacent molecules in the same molecular dimer. The symbol of coupling is determined by the relative orientation of the selected molecular pair's internal transition dipole moment, that is, the J-type is negative and the H-type is positive.^15^ According to our calculation results, both the dimers in B-form and G-form crystals show positive exciton–vibration coupling and the results show a significant increase in the EC value of G-form crystals (Δ*ε* = 27.1 meV) compared to B-form crystals (Δ*ε* = 23.7 meV) as shown in Fig. S11.† These results indicate that both B-form and G-form crystals exhibit typical H-aggregate characteristics. A slight change in packing mode (2%) amplifies the exciton–vibration coupling (14.3%) from B-form to G-form H-aggregates. And the G-form belongs to stronger H-aggregates, which means a lower emission oscillator strength.

**Fig. 4:**
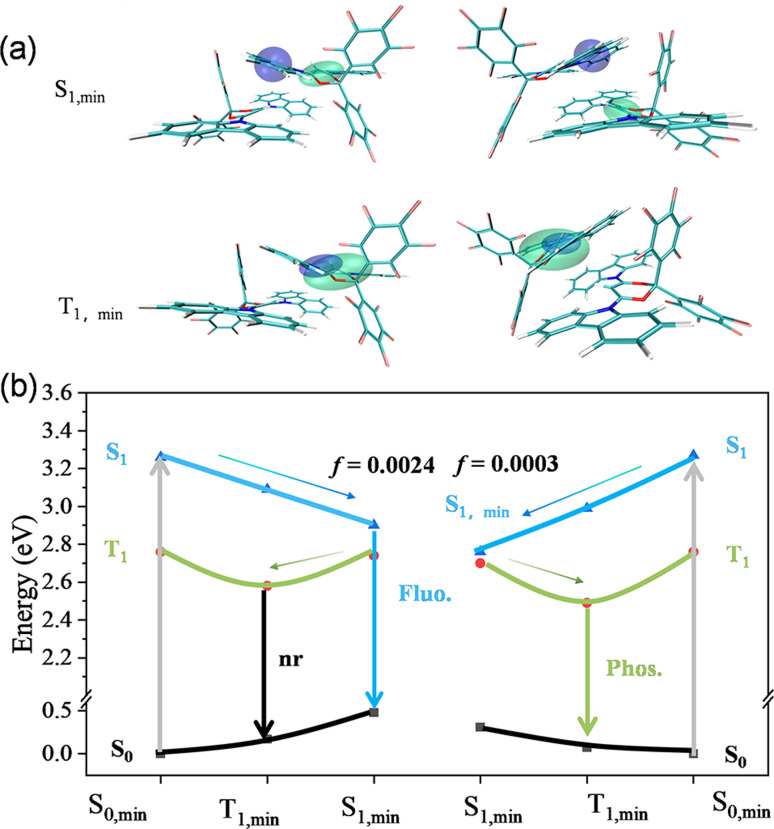
(a) Analysis of the excited state properties of B-form (left) and G-form (right) crystals using images of Cele (green) and Chole (blue) (isovalue = 0.001). (b) Schematic diagram of the B-form fluorescence emission and G-form RTP emission.

To model the effect of a solid-state environment, the quantum mechanics/molecular mechanics (QM/MM) method was used by embedding a dimer (QM at the B3LYP-D3/6-31g(d) level) in the crystal lattice (with 52 surrounding molecules in total as the rigid MM part) as shown in Fig. S12.†Fig. 4a presents the electron–hole distribution wave functions of the optimized minima of S_1, min_ and T_1, min_ in both B- form and G-form crystals . For the excited states of S_1, min_ in B-form crystals, the holes and electrons are distributed in the same molecule in a dimer, indicating a local excited (LE) transition. However, the overlap between HOMO and LUMO orbitals is quite small, resulting in a small Δ*E*_ST_ value of 0.32 eV, which is consistent with the experimental value of 0.32 eV. For G-form crystals, the hole and electron excited states of S_1, min_ are distributed in different molecules in the dimer with no overlap between HOMO and LUMO orbitals, exhibiting a stronger CT state characteristic. The G-form crystals also have a very small Δ*E*_ST_ value of 0.26 eV, which is consistent with the experimental value of 0.22 eV. For the T_1, min_ excited state of dimers in B-form and G-form crystals, the holes and electrons are equally delocalized on a carbazole group, indicating both have LE state characteristics. Obviously, the difference of G-form crystals in characteristics between the S_1_ and T_1_ excited states was larger, which is more favourable for the ISC process to occur and advantageous for phosphorescence emission.

Generally, H aggregates inherently have a relatively low emission oscillator strength. The calculated emission oscillator strength (*f*) of S_1_ states for the B-form dimer is 0.0024, much larger than 0.0003 for the G-form dimer, which ensures a relatively rapid radiative rate and therefore fluorescence as shown in Fig. 4. However, the smaller oscillator strength of the G-form dimer can suppress fluorescence, which facilitates the ISC process and enhances phosphorescence. Combined with the stronger intermolecular interaction in G-form crystals, the RTP is mainly due to the enhanced ISC process to generate the excited triplet state and restrict non-radiative transitions through the multiple intermolecular interactions to stabilize the triplet excitons.

**Fig. 5:**
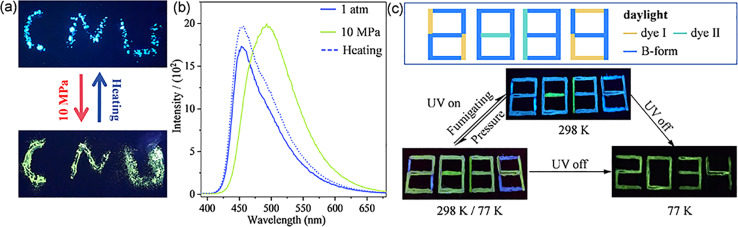
(a and b) Photos (a) and PL spectra (b) of the B-form powder during the pressure–heating process. (c) Information encryption photos made with dye I, dye II, and the B-form powder of ArBFO. The UV light illumination is 365 nm.

Encouraged by the entirely different PL mechanisms and the similar molecular conformation and packing modes for the B-form and G-form crystals, the transformation from the B-form to G-form was performed. Generally, under pressure at the GPa level, the twisted molecular conformation might transform to more planar and the packing mode in the lattice might be more closed, which induces the change of emission wavelength and intensity.^16^ As shown in Fig. 5a, the as-synthesized B-form pristine crystalline powder shows a strong blue emission with a maximum emission at 456 nm at atmospheric pressure. Interestingly, as shown in Fig. 5b, after pressing at 10 MPa, the emission colour of the pressed powder showed a distinct change from blue to green with a red-shifted emission maximum to 494 nm. Significantly, these results show that, as piezoceramic luminescent (PCL) materials, ArBFO aggregates are 2–3 orders of magnitude more sensitive than conventional PCL materials to pressure at the GPa level.^17^ Excitingly, after being heated above 130 °C or fumigation with ethanol vapor, the pressed powder recovers to the initial blue colour. The powder X-ray diffraction (PXRD) analysis shown in Fig. S13c† verified the pressure induced transition from B-form to G-form. Moreover, upon treatment with the pressure–heating process, a reversible blue–green colour switch with good reproducibility and photo stability is realized as shown in Fig. S13b and S14†. In addition, it exhibits good stability under different conditions (Fig. S16†). Note that the ArBFO powder encapsulated in the PVA medium still maintains sensitive PCF behaviours after completely drying in air. Compounds I and II (which are not PCF materials) with similar PL emission peak positions to B-form and G-form crystals, respectively, were selected (Fig. S15†), and they were respectively prepared into PVA inks using the same method. Fig. 5c shows the three-component cryptogram drawn using Ink1 (dye II solution), Ink 2 (dye I solution) and Ink 3 (B-form solution) which were dropped on positions 1, 2 and 3, respectively. A number ‘8888’ was prepared (Fig. S11†). Under excitation at 365 nm, the blue emissive number ‘8038’was read. When pressed with fingers, the emission colour of B-form crystals changes while those of II and I films do not change. A green emissive number ‘2884’ was read. Cooling at 77 K, after removing the UV light illumination, the PL pattern became a green number “2034” lasting for 3 s (Fig. S17†). When the pattern was heated at a relatively low temperature of 120 °C, the security label password reverts to blue-emissive “8038” under 365 nm. The system has good reversibility and can achieve good packaging safety and multi-level information encryption ability. More impressively, these PVA inks can be drawn on different flexible substrates, including plastic, paper, tinfoil, and so on, (Fig. S18†) which holds significant potential for great potential applications in the fields of haptic sensors and wearable flexible anticounterfeiting devices.

## Conclusions

In summary, we designed and synthesized ArBFO molecules and cultivated two crystals with entirely different PL mechanisms. A mechanistic study combining X-ray diffraction analysis and theoretical calculations reveals that a tiny change in molecular packing amplifies exciton–vibration coupling from B-form to G-form H-aggregates and enhances the intermolecular interaction, leading to a large transformation from blue fluorescence to green RTP. Owing to the loosely similar packing mode, the transformation between B and G H-aggregates leads to a reversible blue fluorescence-green RTP switch with good reproducibility and photostability when treated with pressure–heating cycles. With the excellent PCL properties and the persistent phosphorescence at 77 K, the applications of multi-level information encryption and anti-counterfeiting have been successfully demonstrated. The results of this research deepen the understanding of the effect of aggregation on the luminescence mechanism and provide a new design guidance for developing smart materials with good performance.

## Data availability

The data (instrumentation, synthetic procedures, structural characterization data, theoretical calculations, and spectroscopic data) that support this article are available in the article itself and its ESI.†

## Author contributions

Y. C. synthesized the molecule and performed the characterization experiments. Z. X. and Y. Y. performed the theoretical calculations. H. F. and Z. X. analysed the experimental results. Z. X. and Y. C. wrote the original draft, and Z. X. reviewed and edited the manuscript. X. Z., H. L., P. W., M. Y. and H. L. helped in the experimental process. All authors discussed the results and commented on the manuscript.

## Conflicts of interest

There are no conflicts to declare.

## Supplementary Material

SC-OLF-D4SC02867H-s001

SC-OLF-D4SC02867H-s002

SC-OLF-D4SC02867H-s003
