# Supplementary material for: Reversible switching from fluorescence to room temperature phosphorescence amplified by exciton-vibration coupling through pressure-induced tiny packing changes
Source: Chem Sci. 2024 Aug 7;15(34):13930–6. doi: 10.1039/d4sc02867h (PMC11317904; doi:10.1039/d4sc02867h)
Supplement: SC-015-D4SC02867H-s002 [file SC-015-D4SC02867H-s002.pdf]

Supplementary Information (SI) for Chemical Science.
This journal is © The Royal Society of Chemistry 2024

# Supporting Information

# Reversibly Switching from Fluorescence to Room Temperature Phosphorescence Amplified by Exciton-Vibration Coupling through Pressure-Induced Tiny Packing Changes

Yangyang Cao, Zhenzhen Xu*, Xinyuqi Zhao, Yong Yang, Haoran Liu, Pingyang Wang, Miao Yu, Hao Li, Hongbing Fu*

Y. Cao, Prof. Z. Xu, X. Zhao, Y. Yang, H. Liu, P. Wang, M. Yu, H. Li, F. Yu, Prof. H. Fu

Beijing Key Laboratory for Optical Materials and Photonic Devices

Department of Chemistry, Capital Normal University

Beijing 100048 (China)

E-mail: xuzhenzhen@cnu.edu.cn; hbfu@cnu.edu.cn.

## General Remarks Reagents and materials

Unless otherwise specified, all reagents used in the experiments were purchased from commercial sources and used without undergoing further purification. For flash column chromatography, silica gel with 200-300 mesh is used.

## Measurement

Analytical thin-layer chromatography (TLC) is performed using precoated TLC plates with silica gel GF-254. Nuclear magnetic resonance ($^1$H NMR) spectra are obtained on a VARIAN 600 MHz spectrometer. Resonance patterns are reported with the notation s (singlet), d (double), t (triplet), q (quartet), and m (multiples). Mass spectra are measured on a high resolution mass spectroscopy (GCT-MS Micromass, UK). The steady-state absorption spectra were measured on a Shimidazu UV-3600 spectrophotometer. The diffuse reflectance absorption spectra were measured on a Hitachi UH-5700 UV-VIS-NIR spectrophotometer. The emission spectra were measured on Horiba FluoroMax-4-NIR spectrophotometers. The powder X-ray diffraction (XRD) patterns were measured by a Bruker D8 advance powder diffractometer operated in the 2θ range from 5° to 30°. The samples were kept at 298 K during data collection. The exact pressure value applied on the sample was determined by the formula "Pressure= Force / Force area". The “Force Area” were the area of all the crystals on the glass substrate. And the “Force” was forming pressure indicator on the digital thrust gauge. The lifetime and time-resolved emission spectra and PL quantum yield (PLQY) are obtained on Edinburgh FLS1000 fluorescence spectrophotometer. The steady-state and delayed spectra, as well as temperature dependent lifetime data of B-form and G-form at 298 K were collected by Edinburgh FLS1000 fluorescence spectrophotometer. The steady-state and delayed spectra of B-form and G-form at 77 K were collected by a streak camera (C10647, Hamamatsu Photonics) upon 400 nm femtosecond laser excitation. The lifetime data at 298 K and 77 K in the emission spectrum of G-form at 460 nm were collected by a streak camera (C10647, Hamamatsu Photonics) upon 400 nm femtosecond laser excitation. Luminescent photos and videos are taken by Huawei Honor 70 under a UV lamp with 365 nm. The theoretical calculation was performed using Gaussion09 D01 package. The structure determined by X-ray analysis was used as the initial geometry. The geometries were optimized at the B3LYP/6-31G* level.

## Synthesis and Characterization

Materials and reagents: carbazole ($C_{12}H_9N$, 98%), malonyl chloride ($C_3H_2Cl_2O_2$, 97%) tetrahydrofuran ($C_4H_8O$ extra dry with molecular sieves, water ≤ 30 ppm, in resealable bottle), and tris(pentafluorophenyl)borane ($C_{18}BF_{15}$, 97%) were purchased from Innochem., and toluene ($C_7H_8$, AR), were purchased from tgchem.

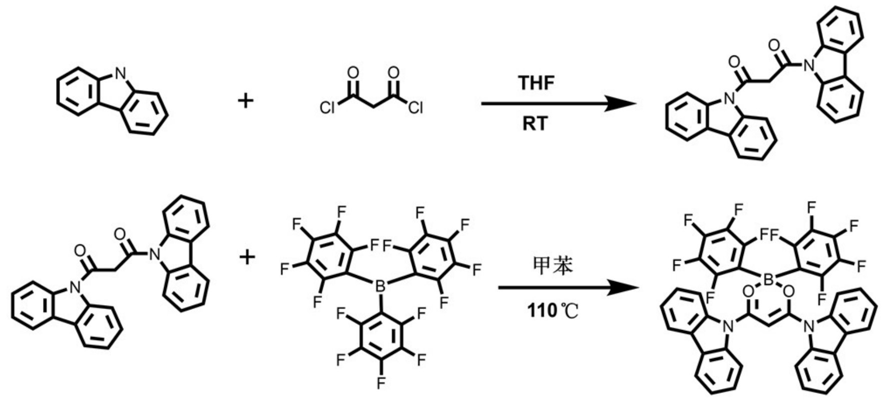


**Figure S1**. The synthetic routes of compound ArBFO.

N1,3-di(9H-carbazol-9-yl)propane-1,3-dione:

9H-carbazole (0.5 g, 3 mmol) and 20 mL dry tetrahydrofuran (THF) were added into a 50 mL two neck flask. Under nitrogen atmosphere, 0.15 mL malonyl dichloride (1.5 mmol) was added slowly. The reaction was then stirred at room temperature for 30 minutes. Then, the reaction solution was concentrated under reduced pressure. The crude product was recrystallization from ethanol/ acetone to afford product as white solid (0.422 g, 70%).

9,9'-(2,2-bis(perfluorophenyl)-2H-1$\lambda^3$,3,2$\lambda^4$-dioxaborinine-4,6-diyl)bis(9H-carbazole)(ArBFO):

The specific synthesis method is documented in Literature 1. 1,3-di(9H-carbazol-9-yl)propane-1,3-dione (0.402 g, 1 mmol), tris(pentafluorophenyl)borane (0.61 g, 1.2 mmol), 10 ml toluene were added in flask. Then the mixture was refluxed for 24 h at 110 °C under nitrogen atmosphere. After the mixture was cooled to room temperature, the solution was extracted with dichloromethane three times. The organic phase was dried with anhydrous sodium sulfate and dichloromethane was removed under reduced pressure. The crude product was further purified by silica gel chromatography to a white powder (0.25 g, 63%). $^1$H NMR (600 MHz, $CDCl_3$) δ (TMS, ppm): 8.24 (d, J = 8.3 Hz, 4H), 8.06 – 8.02 (m, 4H), 7.55 – 7.51 (m, 4H), 7.48 (dd, J = 7.5, 1.0 Hz, 4H), 7.02 (s, 1H). $^{13}$C NMR (101 MHz, $CDCl_3$) δ ppm: 167.36, 138.06, 127.85, 127.46, 125.28, 120.45, 116.36, 80.10.

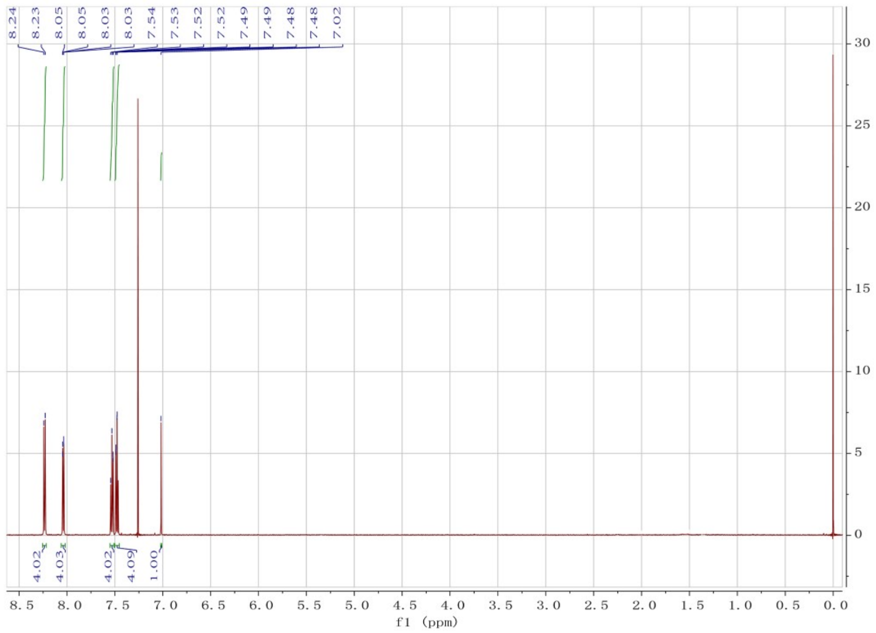


**Figure S2**.1HNMR (600 MHz, $CDCl_3$, 293 K) of compound ArBFO.

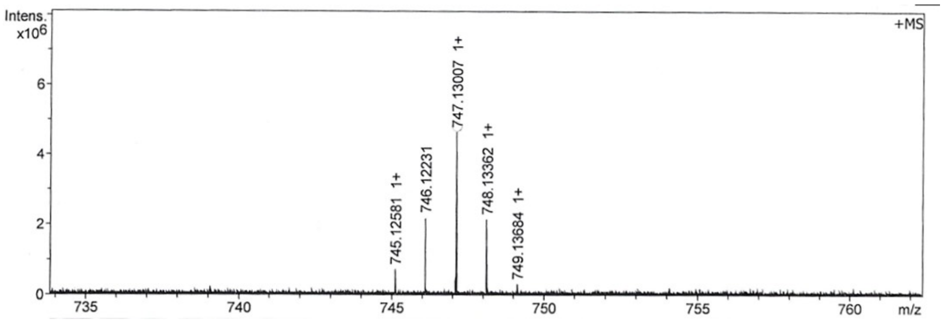


**Figure S3**. HRMS (ESI) m/z [M+Na] $^{+}$ for $C_{39}H_{17}BF_{10}N_2O_2$: 746.37; found: 747.13.

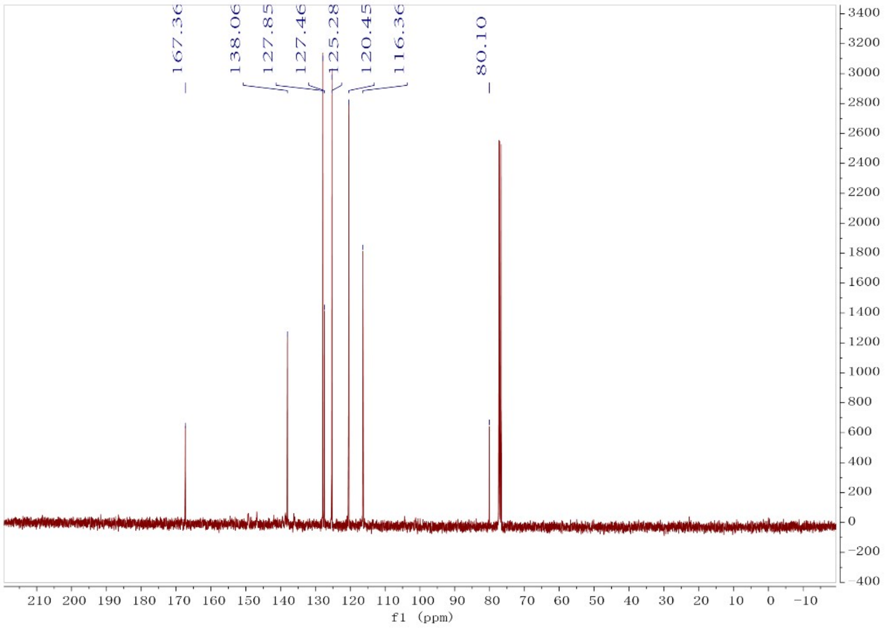


**Figure S4**. $^{13}$C NMR spectra of compound ArBFO.

## Preparation of the crystal

Two kinds of polymorph crystals with high quality and homogeneity were obtained in high throughput under different growth conditions. The color of these crystals differs from green (B-form) to orange (G-form). B-form single crystals with sizes about 3 mm × 1 mm were prepared by the solvent diffusion method at the liquid-liquid interface between hexane and the dichloromethane solution in the 5 mL glass sample at room temperature. G-form single crystals with sizes about 3 mm × 1 mm were prepared by the solvent diffusion method at the liquid-liquid interface between cyclohexane and the dichloromethane solution in the 5 mL glass sample at room temperature.

## Photophysical properties

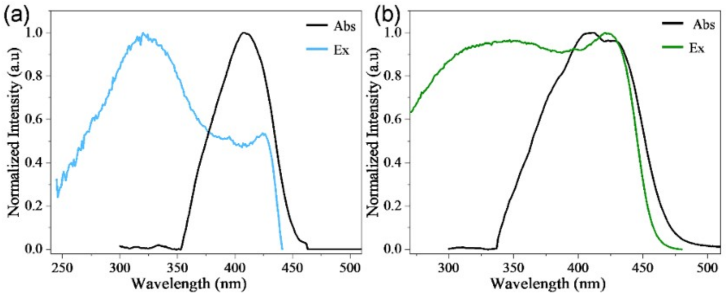


**Figure S5.** Normalized diffuse reflectance absorption and excitation spectra of B-form (a) and G-form crystals (b).

- From the excitation spectra and diffuse reflectance absorption spectra, the excitation spectra of the two crystals are relatively consistent with their respective diffuse reflectance absorption spectra.

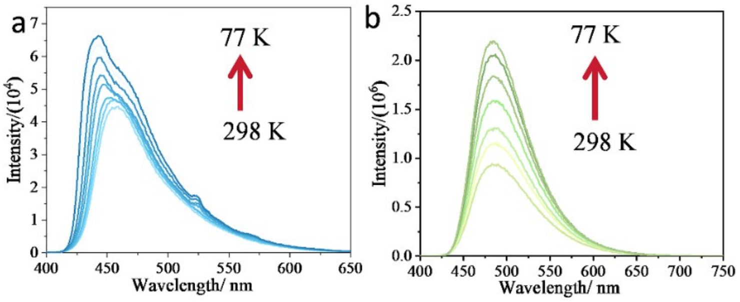


**Figure S6.** Steady-state spectra of B-form crystals (a) and G-form crystals (b).

- We collected the temperature dependent photoluminescence spectra of B-form and G-form crystals. As shown in Figure S4a, at room temperature, the maximum emission peak is displayed at 460 nm, and its intensity increases with decreasing temperature. As shown in Figure S4b, as the temperature decreases, the PL spectrum of the green crystal remains unchanged, with a maximum value of around 499 nm. At the same time, the PL intensity at 77K is significantly enhanced by about 6 times compared to that at 298K.

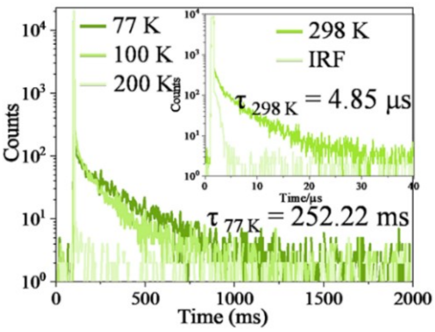


**Figure S7**. Temperature-dependent transient decay spectra of G-form crystals.

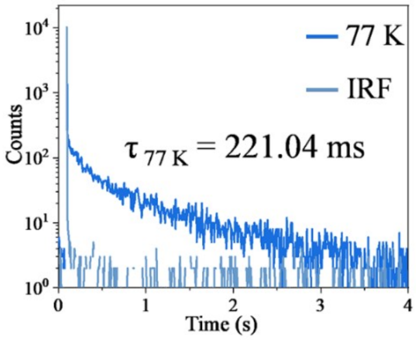


**Figure S8**. PL decay curves of B-form crystals measured at 520 nm in 77K.

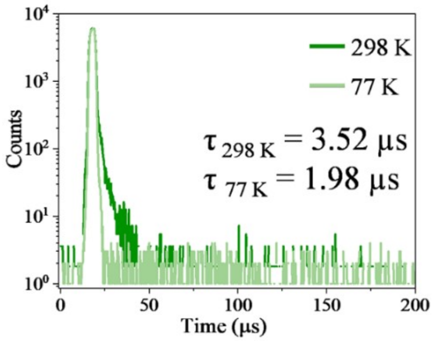


**Figure S9**. Lifetime of G-form with an emission wavelength of 460nm at 298 K and 77 K

- The main emission peak of the steady-state spectrum at 298 K is 499 nm. And the main emission peak of phosphorescence at 77 K is approximately 510 nm, which means the maximum emission wavelength undergoes a slight redshift at low temperature. In addition, the PL and phosphorescence spectra both at 77 K in Figure 2f are different significantly. These results all indicated that the delayed PL at 298 K might not be entirely attributed to phosphorescence. To verify this, the lifetime measurement of different wavelengths in PL spectrum of G-form crystal at 298 K and 77 K were performed. At 298 K, the lifetime of the emission wavelength at 460 nm is 3.52 μs, and at 77 K, the lifetime is decreased to 1.98 μs, exhibiting a trend of decreasing lifetime with the decrease of temperature. This is a typical feature of thermally activated delayed fluorescence (TADF). Therefore, a small amount of TADF component at 460 nm might exist in the spectrum of G-form.

**Table S1**. Summary of the single crystals data of B-form and G-form.

| | ArBFO-B | ArBFO-G |
|---|---|---|
| CCDC | 2336022 | 2336023 |
| Crystal system | triclinic | triclinic |
| Space group | P-1 | P-1 |
| a/Å | 11.1573(5) | 10.9213(3) |
| b/Å | 15.7640(9) | 15.6626(4) |
| c/Å | 20.1788(10) | 20.1705(6) |
| α/° | 77.015(5) | 77.832(2) |
| β/° | 77.796(4) | 78.451(2) |
| γ/° | 81.704(4) | 82.208(2) |
| Volume/Å$^3$ | 3362.7(3) | 3288.74(16) |
| Z | 4 | 4 |
| $\rho_{calc}$g/cm$^3$ | 1.474 | 1.507 |
| Goodness-of-fit on F$^2$ | 1.024 | 1.100 |
| Final R indexes [I>=2σ (I)] | R1 = 0.0449, wR2 = 0.1017 | R1 = 0.1055, wR2 = 0.2766 |
| Final R indexes [all data] | R1 = 0.0655, wR2 = 0.1150 | R1 = 0.1162, wR2 = 0.2805 |

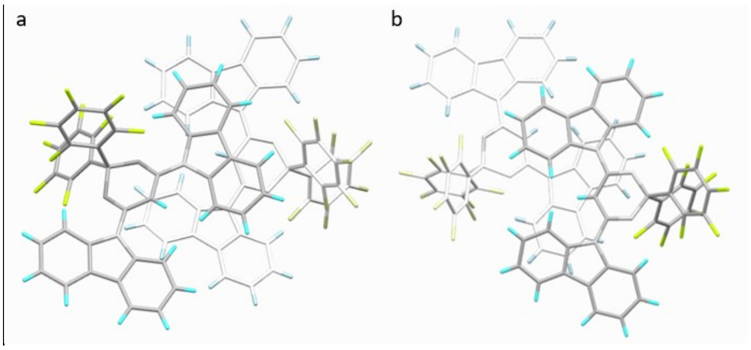


**Figure S10.** The overlap of (a) B-form and (b) and G-form crystal.

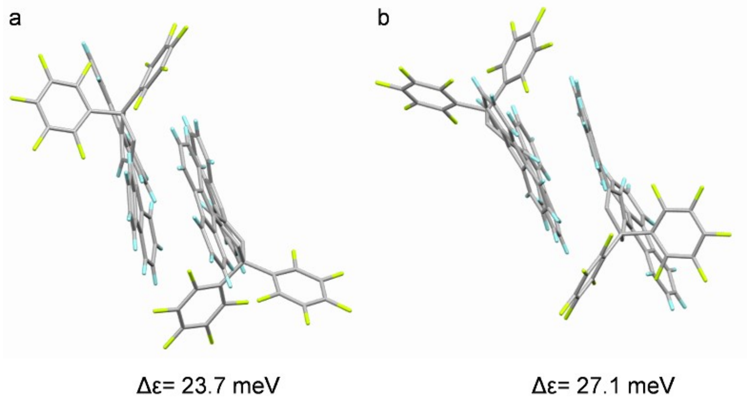


**Figure S11**. Exciton coupling values of (a) B-form (b) and G-form crystal.

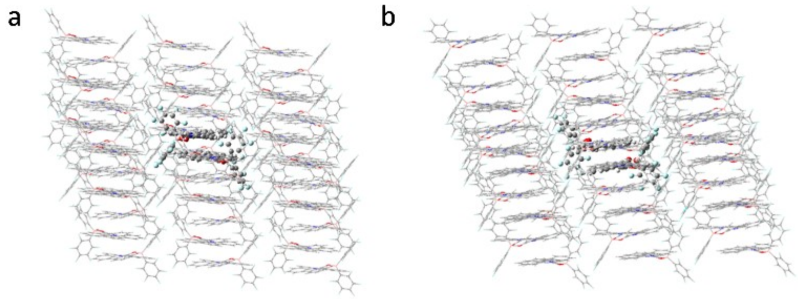


**Figure S12**. Setting of QM/MM model for (a) B-form (b) and G-form crystal.

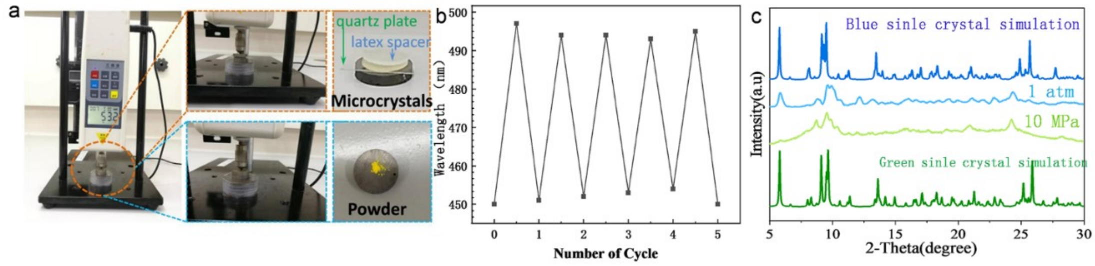


**Figure S13**. (a) Representative photos of the quantitative pressure test by using a digital thrust meter. (b) Cyclic switch of the wavelength and intensity during the pressure - heating process. (c) PXRD patterns of power upon pressure. The simulated results based on the single-crystal data are also included.

- The simulated PXRD results of the G-and B-form crystals data as shown in Figure S10 (c) are almost the same, indicating the same molecular packing. The PXRD analysis of the B-form crystalline powder, and the pressed B-form crystalline powder at 10 MPa were also performed and well matched with the simulated pattern. The slight peaks shift, the differences in intensity and the missing of the partial diffraction peaks in the experimental data as compared to the simulated pattern can be ascribed to the great difference of experiment temperature and the preferred orientation in the crystals with size of mm.

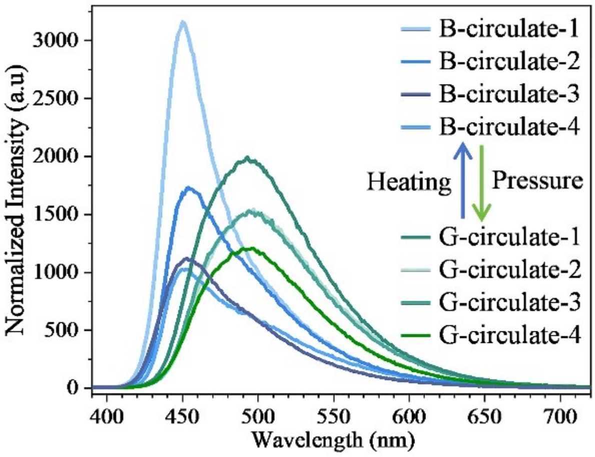


**Figure S14**. Emission spectra of two types of powder crystals during pressure-heating cycle.

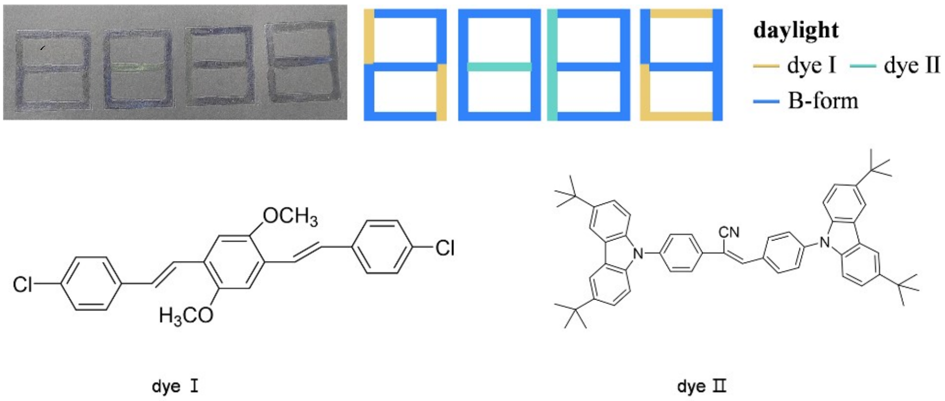


**Figure S15**. The chemical structural formulas of dye I , dye II and the state of dye I, dye II and B-form under daylight.

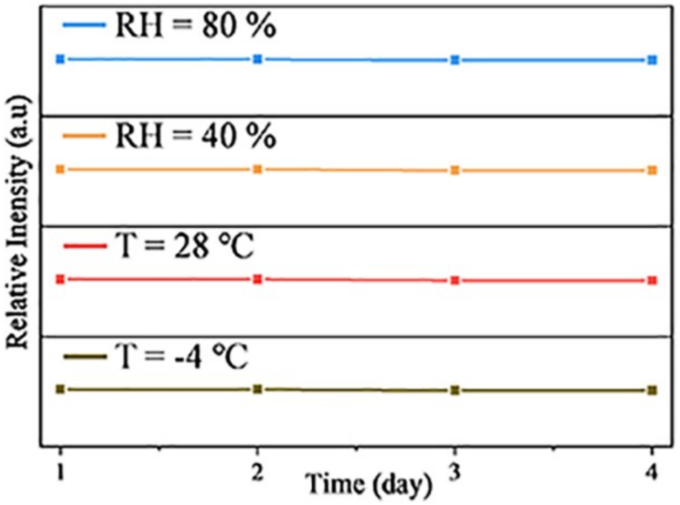


**Figure S16**. The relative PL intensity of ArBFO power for 4 days.

- Stability tests were conducted on ArBFO powder at different temperatures and humidity. The experiment showed that the relative emission spectra of ArBFO powder remained relatively consistent at different temperatures and humidity around 4 days, indicating that ArBFO has excellent stability in different environments and has potential applications in anti-counterfeiting equipment in different environments.

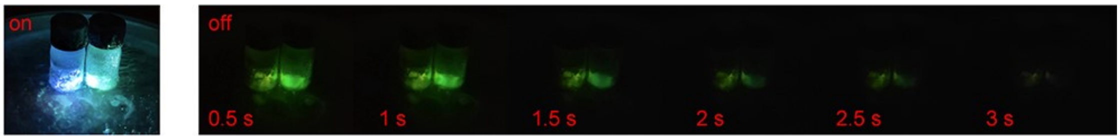


**Figure S17**. Afterglow photos of ArBFO at different times at 77 K

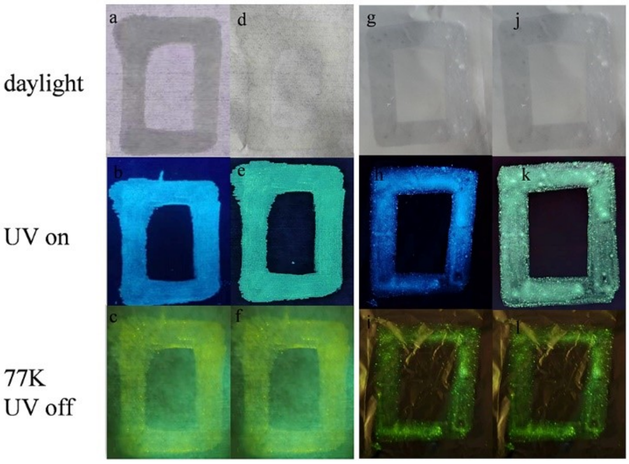


**Figure S18**. (a-f) Images of the word "0" written used the ink on the fabric: under daylight (a, d), UV lamp of 365 nm (b, e) and UV lamp of 365 nm (c, f) in 77 K. (g-l) Images of the word "0" written used the ink on the tinfoil: under daylight (g, j), UV lamp of 365 nm (h, k) and UV lamp of 365 nm (i, l) in 77 K.

- Encouraged by the highly sensitive PCF properties with high emission, the ArBFO B-form power aqueous inks were prepared. The preparation procedure is as follows: after the prepared poly (vinyl alcohol) PVA aqueous solution (0.05 g· $mL^{-1}$) was prepared via dissolving 1 g of PVA in 20 mL of DI water at 80 °C for 2 h. Disperse 10 mg ArBFO powder into 1 mL of the above PVA aqueous solution. The solution was then stirred for 10 min to form a homogeneous ArBFO ink. Write "0" on the filter paper using ArBFO ink, and after crushing, the emission color changes from blue to green.
- Note that the ArBFO microcrystals encapsulated in the PVA medium still maintain the sensitive PCF behaviors after completely drying in air. Drop ArBFO ink onto filter paper to form a uniform film, and after rolling, the emission color changes from blue to green. More impressively, ArBFO ink can be drawn on different flexible substrates, including, fabric, tinfoil, and so on, which shows great potential applications in the field of haptic sensors and wearable flexible anticounterfeiting devices.
- The preparation methods for dye I and dye II water-based inks are the same as those for ArBFO water-based inks.
